# Propofol synergizes with circAPBB2 to protect against hypoxia/reoxygenation‐induced oxidative stress, inflammation, and apoptosis of human cardiomyocytes

**DOI:** 10.1002/iid3.952

**Published:** 2023-08-08

**Authors:** Chenghao Jin, Shunnv Yuan, Longyi Piao, Mingcheng Ren, Qiang Liu

**Affiliations:** ^1^ Department of Anesthesiology Beijing Tongren Hospital, Capital Medical University Beijing China; ^2^ Laboratory Medicine The Affiliated Hospital of Yanbian University Jilin China; ^3^ Department of Oncology Jilin Central Hospital of Jilin University Jilin China; ^4^ Department of Oncology Dandong Central Hospital Dandong Liaoning China

**Keywords:** circAPBB2, DUSP14, miR‐18a‐5p, myocardial injury, propofol

## Abstract

**Background:**

Myocardial injury is the main manifestation of cardiovascular diseases, and previous studies have shown that propofol (PPF) regulates myocardial injury. However, the mechanism of PPF in regulating myocardial injury remains to be further explored. This work aims to analyze the effects of PPF on human cardiomyocyte injury and the underlying mechanism.

**Methods:**

The regulatory and functional role of PPF and circAPBB2 in human cardiomyocyte injury were analyzed using an in vitro hypoxia/reoxygenation (H/R) cell model, which was established by treating human cardiomyocytes (AC16 cells) with H/R. The study evaluated AC16 cell injury by analyzing cytotoxicity, oxidative stress, inflammation and apoptosis of H/R‐induced AC16 cells. Quantitative real‐time polymerase chain reaction was performed to detect circAPBB2, miR‐18a‐5p and dual specificity phosphatase 14 (DUSP14) expression. Protein expression was analyzed by Western blot analysis assay. Dual‐luciferase reporter assay, RNA pull‐down assay and RNA immunoprecipitation assay were performed to identify the associations among circAPBB2, miR‐18a‐5p and DUSP14. Cytotoxicity was investigated by cell counting kit‐8 assay and lactate dehydrogenase activity detection kit. Oxidative stress was evaluated by cellular reactive oxygen species assay kit and superoxide dismutase activity assay kit. The production of tumor necrosis factor‐α and interleukin‐1β was evaluated by enzyme‐linked immunosorbent assays.

**Results:**

The expression of circAPBB2 and DUSP14 was significantly decreased, while miR‐18a‐5p was increased in H/R‐induced AC16 cells when compared with controls. H/R treatment‐induced cytotoxicity, oxidative stress, inflammation and cell apoptosis were attenuated after circAPBB2 overexpression or PPF treatment, whereas these effects were restored by increasing miR‐18a‐5p expression. PPF treatment improved the inhibitory effect of ectopic circAPBB2 expression on H/R‐induced cell injury. MiR‐18a‐5p silencing ameliorated H/R‐induced AC16 damage by interacting with DUSP14. Mechanically, circAPBB2 acted as a miR‐18a‐5p sponge, and miR‐18a‐5p targeted DUSP14 in AC16 cells.

**Conclusion:**

PPF synergized with circAPBB2 to protect AC16 cells against H/R‐induced oxidative stress, inflammation and apoptosis through the miR‐18a‐5p/DUSP14 pathway.

## Graphical abstract

Propofol synergized with circAPBB2 protected AC16 cells against H/R‐induced oxidative stress, inflammation and apoptosis through the miR‐18a‐5p/DUSP14 pathway.

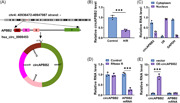

## 1 INTRODUCTION

Cardiovascular disease (CVD) remains the leading cause of human death in the world, and the occurrence of CVD can lead to myocardial ischemia/reperfusion (I/R) injury, resulting in a variety of diseases.^1^ The frequent occurrence of myocardial I/R injury can lead to irreversible injury and even death. Mitochondrial disturbances and the resulting oxidative stress play a vital part in the development of cardiac damage after I/R.^2^ In addition, Physiological and pathological processes such as apoptosis and inflammation are involved in the mechanisms of myocardial I/R injury.^3^ Thus, an in‐depth study of the above pathological mechanisms has important clinical and basic research significance for the prevention and treatment of CVDs.

Circular RNA (circRNA) is identified as a brand‐new star molecule that has a covalently closed ring structure and is abundantly expressed in eukaryotic cells.^4^ The circular molecule does not have a polyadenylated tail and is more resistant to exonuclease treatment. CircRNA can act as a microRNA (miRNA) sponge, translation regulator and protein sponge.^5^ Increasing studies have demonstrated that circRNA participates in the development of CVDs, such as I/R injury, myocardial infarction, and coronary artery disease.^4^ In particular, circ‐NNT expression was upregulated in patients with myocardial infarction and its silencing attenuated myocardial infarction.^6^ CircACR ameliorated myocardial I/R injury through the PTEN induced kinase 1 (PINK1)/FAM65B axis.^7^ CircAPBB2 homologous to mouse circ_0001352 has been identified as a downregulated circRNA in C57BL/6 mice after myocardial I/R injury protocol^8^; however, its role and the underlying mechanism in human cardiomyocyte injury following myocardial I/R have not been reported.

The AMIGEO database shows that some miRNAs including miR‐34a, miR‐545, miR‐33a, miR‐101, miR‐598, miR‐24‐1, miR‐142‐3p and miR‐139‐3p are differentially expressed in the plasma of patients with myocardial infarction.^9^ Therefore, they could be used as targets for diagnosis and treatment of myocardial infarction. MiR‐1 and miR‐133a participate in early cardiogenesis, and miR‐208 and miR‐499 regulate later cardiomyocyte differentiation.^10^ Through bioinformatics analyses, we found that circAPBB2 might act as a miR‐18a‐5p sponge. In addition, a previous study revealed that dual specificity phosphatase 14 (DUSP14), a predicted target of miR‐18a‐5p, protected against myocardial I/R injury.^11^ Whether circAPBB2 regulates myocardial I/R injury through the miR‐18a‐5p/DUSP14 axis remains unknown.

Propofol (PPF) is an intravenous anesthetic commonly used for postoperative sedation and anesthesia induction and maintenance. Pharmacological research has shown that PPF can inhibit the occurrence of inflammation and the production of proinflammatory cytokines.^12^ Convincing evidence also reveals that PPF can exert an antioxidant effect by inducing the expression of oxidative stress biomarkers.^13^ Moreover, PPF can mediate cardioprotection against myocardial I/R injury by various mechanisms such as the miRNA‐451/high mobility group box 1 (HMGB1) pathway^14^ and the AKT/p53 signaling pathway.^15^ So far, there has been no study regarding whether PPF can protect human cardiomyocytes against myocardial I/R injury by combining with circAPBB2. This study was designed to analyze the effects of PPF and circAPBB2 on myocardial I/R injury using a hypoxia/reoxygenation (H/R)‐induced human cardiomyocyte model. Moreover, we analyzed whether the regulation of circAPBB2 in H/R‐induced human cardiomyocyte injury involved the circAPBB2/miR‐18a‐5p/DUSP14 pathway.

## 2 MATERIALS AND METHODS

### 2.1 Cell culture

Human cardiomyocytes (AC16) were obtained from EK‐Bioscience and cultured in Dulbecco's modified Eagle's medium (DMEM; #L110KJ; EK‐Bioscience) with 10% fetal bovine serum (FBS) (#C2056‐1A, EK‐Bioscience) and 1% penicillin/streptomycin (#PH1513, Phygene) at 37°C in an atmosphere containing 5% CO_2_.

### 2.2 H/R protocol and PPF treatment

AC16 cells were maintained in DMEM at 37°C for 24 h and then washed with phosphate buffered solution. The medium was replaced with serum‐free DMEM, and the cells were cultured for another 12 h. Subsequently, the AC16 cells were exposed to an atmosphere buffered with 1% O_2_, 5% CO_2_ and 94% N_2_ for 4 h. The cells were incubated in DMEM containing 10% FBS in an incubator supplemented with 95% O_2_ and 5% CO_2_ for reoxygenation. After being maintained for 4 h, the cells were used for further studies. Cells cultured in incubators buffered with 95% O_2_ and 5% CO_2_ were used as controls.

AC16 cells were exposed to a series of concentrations of PPF (50, 100, and 150 μM; #A18293; Adooq) for 2 h and then subjected to H/R treatment and reoxygenation treatment.

### 2.3 MiRNAs, plasmids, and transfection

MiR‐18a‐5p mimics (5'‐UAAGGUGCAUCUAGUGCAGAUAG‐3') and inhibitors (5'‐CUAUCUGCACUAGAUGCACCUUA‐3'), the overexpression plasmid of circAPBB2 (built using the pCD5‐ciR vector; OE‐circAPBB2), the small interfering RNA against DUSP14 (5'‐GAGACATAGGAGGCATTGCTCAAAT‐3'), and the corresponding controls were all synthesized by Ribobio Co., Ltd. Then, AC16 cells were used for the transient transfection of the above oligonucleotides and plasmids in line with the guidebook of Lipofectamine 2000 (#12566014, Thermo Fisher). The transfection efficiency was detected 48 h following transfection.

### 2.4 RNA isolation and quantification

RNA isolation reagents (#DP419; Tiangen) were used for RNA preparation of AC16 cells according to the manufacturer's protocol. Nuclear and cytoplasmic RNAs were isolated after nucleocytoplasmic separation following the instruction of the PARIS™ Kit (#AM1921; Thermo Fisher). Reverse transcription processes were performed using the cDNA Reverse Transcription Kit (#KR116; Tiangen) and miRNA RT reagents (#4366597; Thermo Fisher) according to the guidebooks. Quantitative real‐time polymerase chain reaction (qRT‐PCR) was performed on 7500 Real‐time PCR System (Applied Biosystems) with SYBR Green PCR Master Mix (#FP207; Tiangen) in accordance with the guidebooks. The qRT‐PCR was cycled at 95°C for 60 s, 40 cycles at 95°C for 5 s, and at 60°C for 15 s. GAPDH and U6 were used to normalize circAPBB2/DUSP14 and miR‐18a‐5p expression, respectively, through the 2^‐∆∆Ct^ method. The primer sequences are shown in Table 1.

**Table 1:** **Primer sequences used for qRT‐PCR**.

Name	size	GenBank Accession numbers	Annealing temperatures		Primers for PCR (5'−3')
circAPBB2	173 bp	hsa_circ_0008453(circBase ID)	55°C	Forward	AGTGACCCAGAAGCCAAGATATA
				Reverse	AGAACCCTGGAGATCTGCTG
APBB2	185 bp	NM_001166050.2	56°C	Forward	GATCTCTTTGGGCTGCCTGA
				Reverse	AGCCCATAGCGACAGTCAAC
miR‐18a‐5p	67 bp	MIMAT0000072 (miRBase ID)	55°C	Forward	GGTTCGAGGTGTGCGGAAATG
				Reverse	GTCGTATCCAGTGCAGGGTC
DUSP14	97 bp	NM_007026.4	56°C	Forward	GCGCCGCGCCGATTT
				Reverse	CCTCAAGAGTCCAGTGTGCG
GAPDH	132 bp	NM_002046.7	55°C	Forward	CAAATTCCATGGCACCGTCA
				Reverse	GACTCCACGACGTACTCAGC
U6	94 bp	NR_104084.1	55°C	Forward	CTCGCTTCGGCAGCACA
				Reverse	AACGCTTCACGAATTTGCGT

### 2.5 RNase R treatment

As instructed,^16^ 2 μg RNA from AC16 cells was maintained in a buffer added with RNase R (#S33294; Yuanye Bio‐Technology) as well as RNase R reaction solution at 37°C for 30 min. For the control group, DEPC‐treated water was used to treat cells. Linear APBB2 was utilized as an internal control.

### 2.6 Dual‐luciferase reporter assay

The sequences of circAPBB2 and DUSP14 3'UTR with miR−18a‐5p‐binding sites were amplified by polymerase chain reaction and used to build wild‐type (WT) reporter vectors (WT‐circAPBB2 and WT‐DUSP14 3'UTR). The miR‐18a‐5p‐binding sites in circAPBB2 and DUSP14 3'UTR were mutated to generate mutant (MUT) reporter vectors (MUT‐circAPBB2 and MUT‐DUSP14 3'UTR). The above reporter plasmids were cotransfected with miR‐18a‐5p mimics or miR‐NC into AC16 cells. After 48 h, Double‐Luciferase Reporter Assay reagents (#D0011; Solarbio) were utilized to assess the luciferase activities of AC16 cells. In brief, the media were removed and cells were incubated with Lysis Buffer for 15 min. Samples, Firefly Luciferase Assay Buffer, and Renilla Luciferase Assay Buffer were added into test wells in sequence, and luciferase activity of each sample was analyzed using GloMax® 20/20 Reporter Gene Assay System (Promega).

### 2.7 RNA immunoprecipitation assay (RIP)

As instructed,^17^ AC16 cell samples were harvested, and the cell samples were lysed by RIP lysis buffer (#P1005; Beyotime). Cell lysates were maintained in buffers with magnetic beads according to the guidebook of Magna RNA immunoprecipitation kit (#17‐700; Millipore), which were incubated with anti‐AGO2 antibody (#ab186733; Abcam) or anti‐IgG antibody (#ab109489; Abcam) in advance. After 24 h, the coprecipitated circAPBB2 was examined by qRT‐PCR.

### 2.8 RNA pull‐down assay

WT and mutant miR‐18a‐5p biotin (WT‐bio‐miR‐18a‐5p and MUT‐bio‐miR‐18a‐5p) and their negative controls (bio‐miR‐NC) were provided by Sangon. AC16 cells transfected with WT‐bio‐miR‐18a‐5p, MUT‐bio‐miR‐18a‐5p and bio‐miR‐NC were harvested and sonicated. The lysates were incubated with magnetic beads (#65801D; Invitrogen) to generate probe‐coated beads. After 4 h, the combined circAPBB2 and DUSP14 RNA in the complex were purified for qRT‐PCR analysis.^18^

### 2.9 Western blot analysis assay

NP‐40 lysis reagent (Beyotime) was used to extract proteins. After their concentration detection using NanoDrop 2000 spectrophotometer, the proteins were loaded onto bis‐tris‐acrylamide gels and transferred onto nitrocellulose membranes, followed by blocking with freshly prepared 5% skim milk powder for 2 h. The membranes were incubated with primary antibodies specific to DUSP14 (#bsm‐51562M; 1:800, Bioss), B‐cell lymphoma‐2 (Bcl‐2; #bsm‐52022M; 1:500, Bioss), cleaved caspase 3 (#PA5‐114687; 1:2000, Thermo Fisher), cleaved poly [ADP‐ribose] polymerase (PARP) (#bsm‐52408R; 1:2000, Bioss), and GAPDH (#bsm‐33033M; 1:2000, Bioss) overnight at 4°C. The membranes were incubated with secondary antibodies (#31430 and #65‐6120; Thermo Fisher) and visualized using enhanced chemiluminescence solution (#P0018S; Beyotime). The density of the band was quantified by Quantity One analytic software.

### 2.10 Cell viability assay

Briefly, AC16 cells were maintained in 96‐well plates for 48 h after a series of treatments. The cells were treated according to the experimental requirements of cell counting kit‐8 (CCK‐8; #C0037; Beyotime). The cells were exposed to 10 μL of CCK‐8 reagent for another 4 h at 37°C. Finally, the OD values of all samples were examined with an RNE90002 microplate reader (REAGEN). The absorbance of each experimental group was measured at 450 nm.

### 2.11 Lactate dehydrogenase (LDH) analysis

LDH assay kit (#C0016; Beyotime) was used to determine LDH activity in AC16 cells with a series of treatments. Briefly, AC16 cells were maintained in 96‐well plates and then cell supernatants were removed. LDH release reagent was diluted using phosphate buffered solution and added into each well. The cells were cultured for another 1 h in an incubator, and the cell supernatants were harvested. Then, cell supernatants were incubated with LDH detection solution for 30 min. Finally, the OD value of each well was examined with an RNE90002 microplate reader (REAGEN).

### 2.12 Reactive oxygen species (ROS) and superoxide dismutase (SOD) measurement

ROS and SOD contents in AC16 cells were measured by cellular ROS assay kit (#ab113851; Abcam) and SOD activity assay kit (#ab65354; Abcam), respectively, in accordance with the manufacturer's procedures. Finally, these samples were examined using an RNE90002 microplate reader (REAGEN).

### 2.13 Enzyme‐linked immunosorbent assay (ELISA)

AC16 cells with various treatments were subjected to lysis using RIPA lysis buffer, and cell supernatant was collected. TNF‐a and interleukin‐1β (IL‐1β) levels in the collected samples were examined in line with the instruction of available ELISA kits (#PT518 and #PI305; Beyotime). Eventually, the OD values of all samples were determined with an RNE90002 microplate reader (REAGEN).

### 2.14 Flow cytometry

AC16 cell apoptosis was analyzed following the instruction of an Annexin V‐FITC/PI Apoptosis kit (#CA1020; Solarbio). In brief, cells were harvested and washed with ice‐cold phosphate buffer solution two times. The cells were suspended in 500 μL of binding buffer added with 5 μL of Annexin V‐FITC (Solarbio) and 10 μL of PI (Solarbio) in the dark at room temperature for 15 min. Attune^TM^ NxT flow cytometer (Parameter: CV < 3%, Fluorescence detection sensitivity: 65,000 cells/s, Detection resolution: 12.5−1000 μL/min, Cell analysis speed: ≤30 MESF; Thermo Fisher) was applied to quantify cell apoptosis.

### 2.15 Statistical analysis

All experiments were performed in triplicate. Data analysis was performed using GraphPad Prism software. Results are presented as mean ± SD. Significant differences were determined using Student's *t*‐tests or analysis of variance with Tukey's test. *p* < .05 indicated a statistical significance.

## 3 RESULTS

### 3.1 CircAPBB2 expression was downregulated in H/R‐stimulated AC16 cells

**Figure 1:**
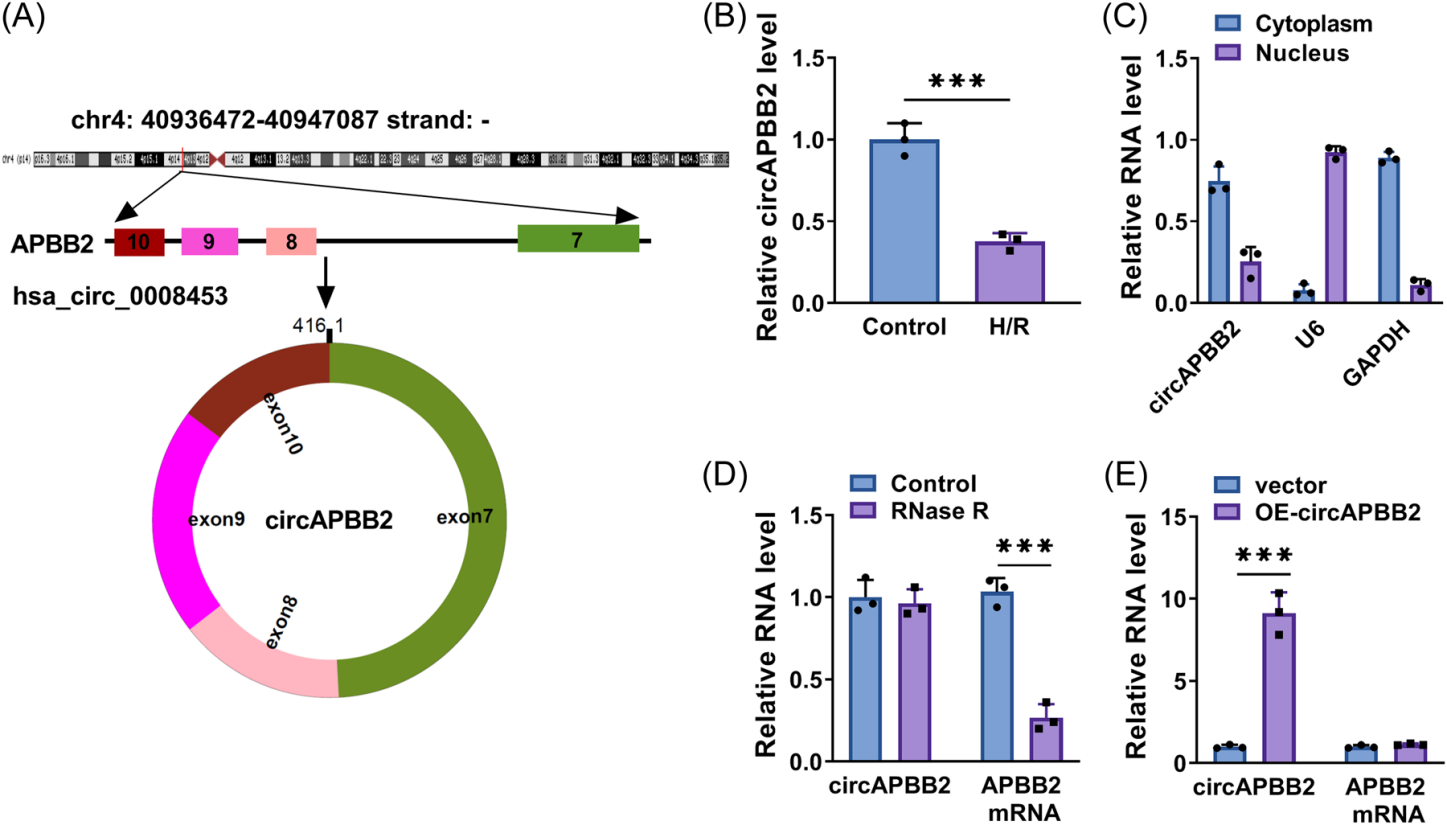
CircAPBB2 expression was downregulated in H/R‐stimulated AC16 cells. (A) The schematic illustration showing the generation of circAPBB2 by the cyclization of the exons 7‐10 of the APBB2 gene. (B) CircAPBB2 expression was analyzed by qRT‐PCR in AC16 cells after treatment with hypoxia or normal oxygen (*N* = 3). (C) Subcellular fractionation location assay was performed to determine the location of circAPBB2 in AC16 cells (*N* = 3). (D) RNase R treatment assay was used to identify the structural stability of circAPBB2 (*N* = 3). (E) The efficiency of circAPBB2 overexpression was analyzed by qRT‐PCR in AC16 cells (*N* = 3). ***p < .001. Significant differences were determined using Student's *t*‐tests.

CircAPBB2 (circRNA_ID: hsa_circ_0008453) was located on the chr4:40936472‐40947087 strand:‐ and was formed by the cyclization of the exons 7‐10 of the APBB2 (Figure 1A). CircAPBB2 expression was decreased in H/R‐stimulated AC16 cells when compared with the control group (Figure 1B). Subsequently, the results showed that circAPBB2 was mainly expressed in the cytoplasm, as presented in Figure 1C. In addition, RNase R treatment significantly downregulated linear APBB2 expression, whereas it had no significant effect on circAPBB2 expression (Figure 1D). Further, the results showed that the transfection with OE‐circAPBB2 significantly increased circAPBB2 expression but not that of APBB2 in AC16 cells (Figure 1E), suggesting the high efficiency of circAPBB2 overexpression in AC16 cells.

### 3.2 CircAPBB2 promoted DUSP14 expression by binding to miR‐18a‐5p

**Figure 2:**
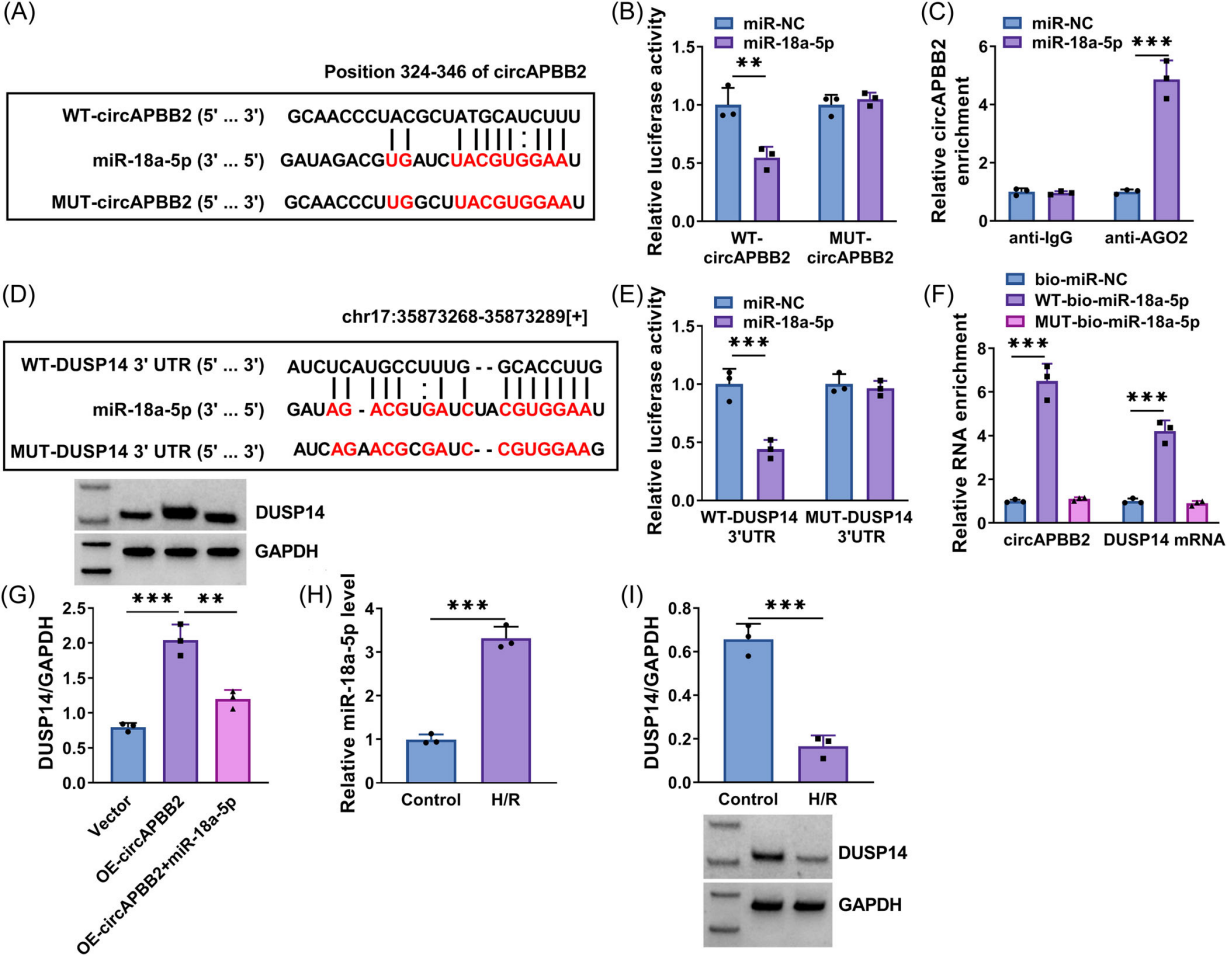
CircAPBB2 promoted DUSP14 expression by binding to miR‐18a‐5p. (A) The schematic illustration showing circAPBB2‐binding sites for miR‐18a‐5p. (B, C) Dual‐luciferase reporter assay and RIP assay were performed to determine the association of circAPBB2 and miR‐18a‐5p (*N* = 3). (D) The schematic diagram showing miR‐18a‐5p‐binding sites for DUSP14. (E) Dual‐luciferase reporter assay confirmed that miR‐18a‐5p targeted DUSP14 (*N* = 3). (F, G) RNA pull‐down assay and Western blot analysis assay were used to demonstrate that circAPBB2 regulated DUSP14 expression by binding to miR‐18a‐5p (*N* = 3). (H, I) MiR‐18a‐5p expression was analyzed by qRT‐PCR and DUSP14 protein expression was detected by Western blot analysis assay in AC16 cells after treatment with hypoxia or normal oxygen (*N* = 3). **p < .01 and ***p < .001. Significant differences were determined using Student's *t*‐tests in (B, C, E, H, I) and determined using one‐way analysis of variance with Tukey's test in (F and G).

As shown in Figure 2A, circAPBB2 contained the complementary sites of miR‐18a‐5p. Subsequently, the dual‐luciferase reporter assay showed that miR‐18a‐5p introduction significantly inhibited the luciferase activity of WT‐circAPBB2 but not that of MUT‐circAPBB2 in AC16 cells (Figure 2B). Meanwhile, the RIP assay revealed that circAPBB2 expression was significantly increased after transfection with miR‐18a‐5p mimics in the anti‐AGO2 group rather than in the anti‐IgG group (Figure 2C). The above data indicated that circAPBB2 bound to miR‐18a‐5p. The binding sites of miR‐18a‐5p for DUSP14 3'UTR are presented in Figure 2D. Similarly, we confirmed that miR‐18a‐5p targeted DUSP14 by the dual‐luciferase reporter assay. For instance, miR‐18a‐5p overexpression significantly reduced the luciferase activity of WT‐DUSP14 3'UTR, whereas it did not affect the luciferase activity of MUT‐DUSP14 3'UTR (Figure 2E). Moreover, we found that circAPBB2 and DUSP14 were significantly enriched in the WT‐bio‐miR‐18a‐5p group rather than in the MUT‐bio‐miR‐18a‐5p group (Figure 2F). Comparatively, ectopic circAPBB2 expression contributed to DUSP14 production, but the effect was attenuated after the cotransfection of OE‐circAPBB2 and miR‐18a‐5p (Figure 2G). Further, H/R treatment increased miR‐18a‐5p expression but inhibited DUSP14 expression, as presented in Figures 2H,I.

### 3.3 CircAPBB2 overexpression ameliorated H/R‐induced myocardial cell oxidative stress, inflammation, and apoptosis by regulating miR‐18a‐5p

**Figure 3:**
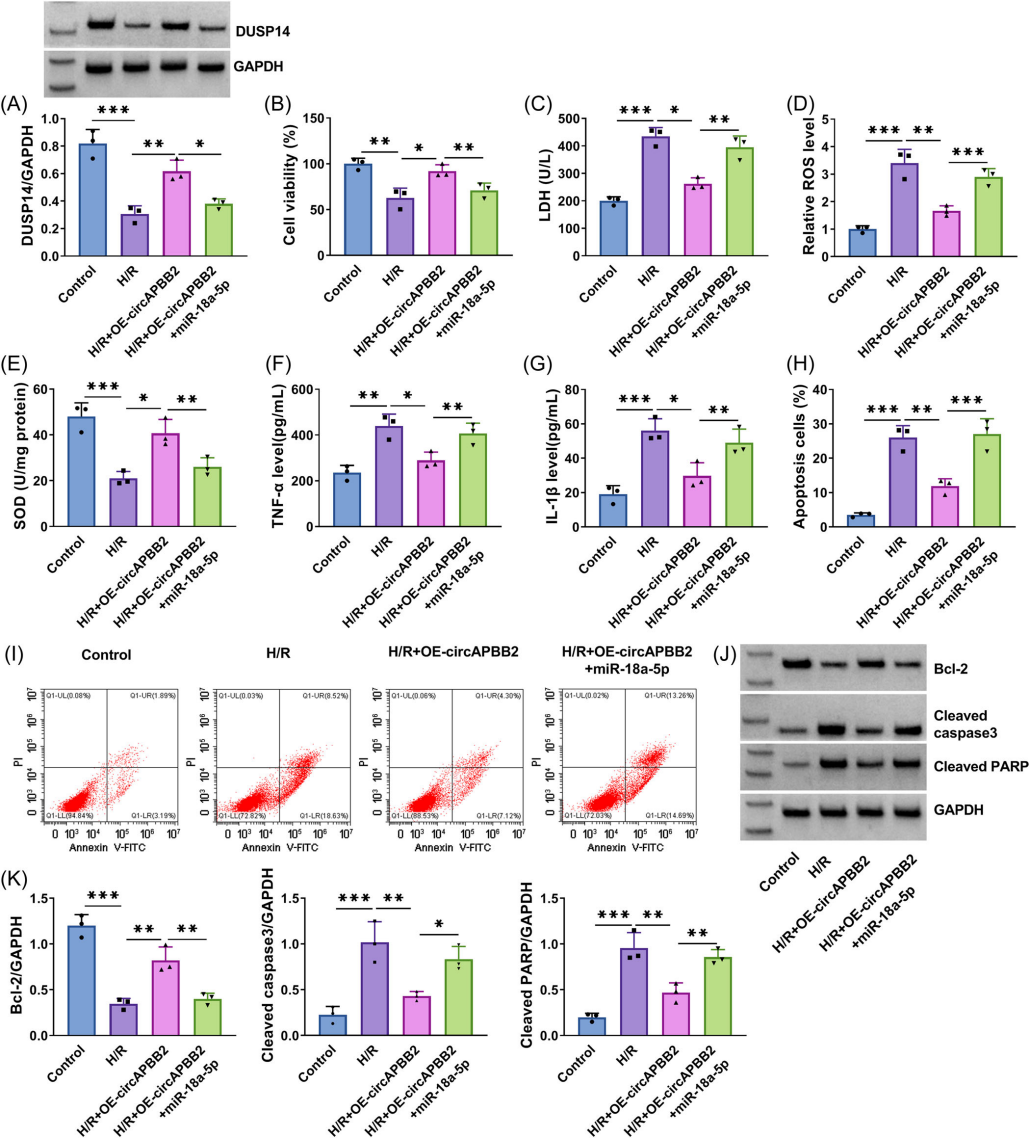
CircAPBB2 overexpression ameliorated H/R‐induced myocardial cell injury by regulating miR‐18a‐5p. AC16 cells were divided into H/R group, H/R + OE‐circAPBB2 group, H/R + OE‐circAPBB2 group+miR‐18a‐5p group, with AC16 cells with normal treatment as controls. DUSP14 protein expression was analyzed by Western blot analysis assay (A) (*N* = 3). Cell viability was assessed by CCK‐8 assay (B) (*N* = 3). LDH, ROS and SOD levels were detected by commercial detection kits (C−E) (*N* = 3). TNF‐α and IL‐1β levels were quantified by ELISAs (F, G) (*N* = 3). Cell apoptosis was quantified by flow cytometry assay (H, I) (*N* = 3). Cleaved caspase 3, cleaved PARP and Bcl‐2 expression were detected by Western blot analysis (J, K) (*N* = 3). *p < .05, **p < .01 and ***p < .001. Significant differences were determined using one‐way analysis of variance with Tukey's test. Bcl‐2, B‐cell lymphoma‐2; CCK‐8, cell counting kit‐8; H/R, hypoxia/reoxygenation; IL‐1β, interleukin‐1β; LDH, Lactate dehydrogenase; ROS, reactive oxygen species; SOD, superoxide dismutase; TNF‐α, tumor necrosis factor‐α.

The results first showed that H/R treatment‐induced inhibition of DUSP14 protein expression was relieved by increasing circAPBB2 expression, whereas the effect was restored when miR‐18a‐5p expression was increased (Figure 3A). Subsequently, H/R treatment‐induced inhibition of cell viability was attenuated after circAPBB2 overexpression, but the effect was rescued by increasing miR‐18a‐5p expression (Figure 3B). As shown in Figure 3C−E, H/R treatment‐induced promoting effects on LDH and ROS production and inhibitory effect on SOD level were attenuated when circAPBB2 expression was increased; however, these effects were restored by miR‐18a‐5p mimics. In addition, the results showed that H/R treatment‐induced increases of tumor necrosis factor‐α (TNF‐α) and IL‐1β levels were relieved by ectopic circAPBB2 expression, but the effect was rescued after transfection with miR‐18a‐5p mimics (Figure 3F,G). Comparatively, the enforced circAPBB2 expression relieved the promoting effects of H/R stimulation on AC16 cell apoptosis and cleaved caspase 3 and cleaved PARP expression and its inhibitory effect on Bcl‐2 expression, but these effects were rescued by miR‐18a‐5p mimics (Figure 3H−K).

### 3.4 MiR‐18a‐5p silencing attenuated H/R‐induced myocardial cell oxidative stress, inflammation, and apoptosis by regulating DUSP14

**Figure 4:**
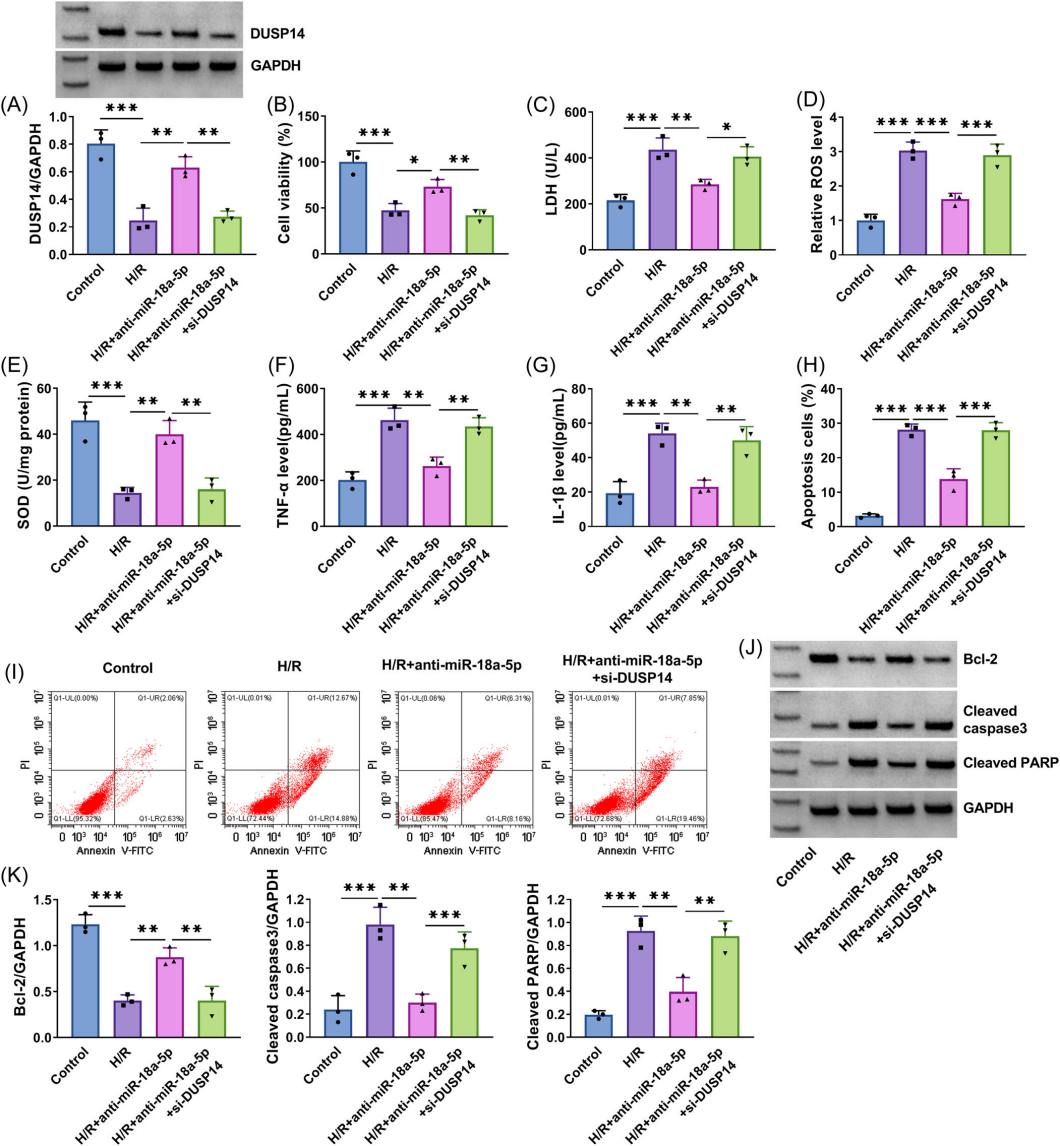
MiR‐18a‐5p depletion ameliorated H/R‐induced myocardial cell injury by regulating DUSP14. AC16 cells were divided into H/R group, H/R+anti‐miR‐18a‐5p group, H/R+anti‐miR‐18a‐5p+si‐DUSP14 group, with AC16 cells with normal treatment as controls. DUSP14 protein expression was analyzed by Western blot analysis assay (A) (*N* = 3). Cell viability was assessed by CCK‐8 assay (B) (*N* = 3). LDH, ROS, and SOD levels were detected by commercial detection kits (C‐E) (*N* = 3). TNF‐α and IL‐1β levels were quantified by ELISAs (F and G) (*N* = 3). Cell apoptosis was quantified by flow cytometry assay (H and I) (*N* = 3). Cleaved caspase 3, cleaved PARP and Bcl‐2 expression were detected by Western blot analysis (J, K) (*N* = 3). *p < .05, **p < .01 and ***p < .001. Significant differences were determined using one‐way analysis of variance with Tukey's test. Bcl‐2, B‐cell lymphoma‐2; CCK‐8, cell counting kit‐8; H/R, hypoxia/reoxygenation; IL‐1β, interleukin‐1β; LDH, Lactate dehydrogenase; ROS, reactive oxygen species; SOD, superoxide dismutase; TNF‐α, tumor necrosis factor‐α.

As presented in Figure 4A, H/R treatment‐induced inhibition of DUSP14 protein expression was relieved by decreasing miR‐18a‐5p expression, whereas the effect was restored when DUSP14 expression was inhibited. Then, H/R treatment‐induced inhibition in cell viability was attenuated after miR‐18a‐5p silencing, but the effect was rescued after transfection with si‐DUSP14 (Figure 4B). The promoting effects on LDH and ROS production and the inhibitory effect on SOD activity induced by H/R treatment were attenuated when miR‐18a‐5p expression was decreased; however, the effect was restored by DUSP14 knockdown (Figure 4C−E). The results also revealed that H/R treatment‐induced increases of TNF‐α and IL‐1β levels were relieved by miR‐18a‐5p inhibitors, but the effect was rescued after transfection with si‐DUSP14 (Figure 4F,G). In addition, miR‐18a‐5p inhibitors relieved the promoting effects of H/R stimulation on AC16 cell apoptosis and cleaved caspase 3 and cleaved PARP expression and its inhibitory effect on Bcl‐2 expression, whereas miR‐18a‐5p inhibitor‐induced effects were rescued after DUSP14 silencing (Figure 4H−K).

### 3.5 PPF treatment protected against H/R‐induced myocardial cell injury by regulating miR‐18a‐5p

**Figure 5:**
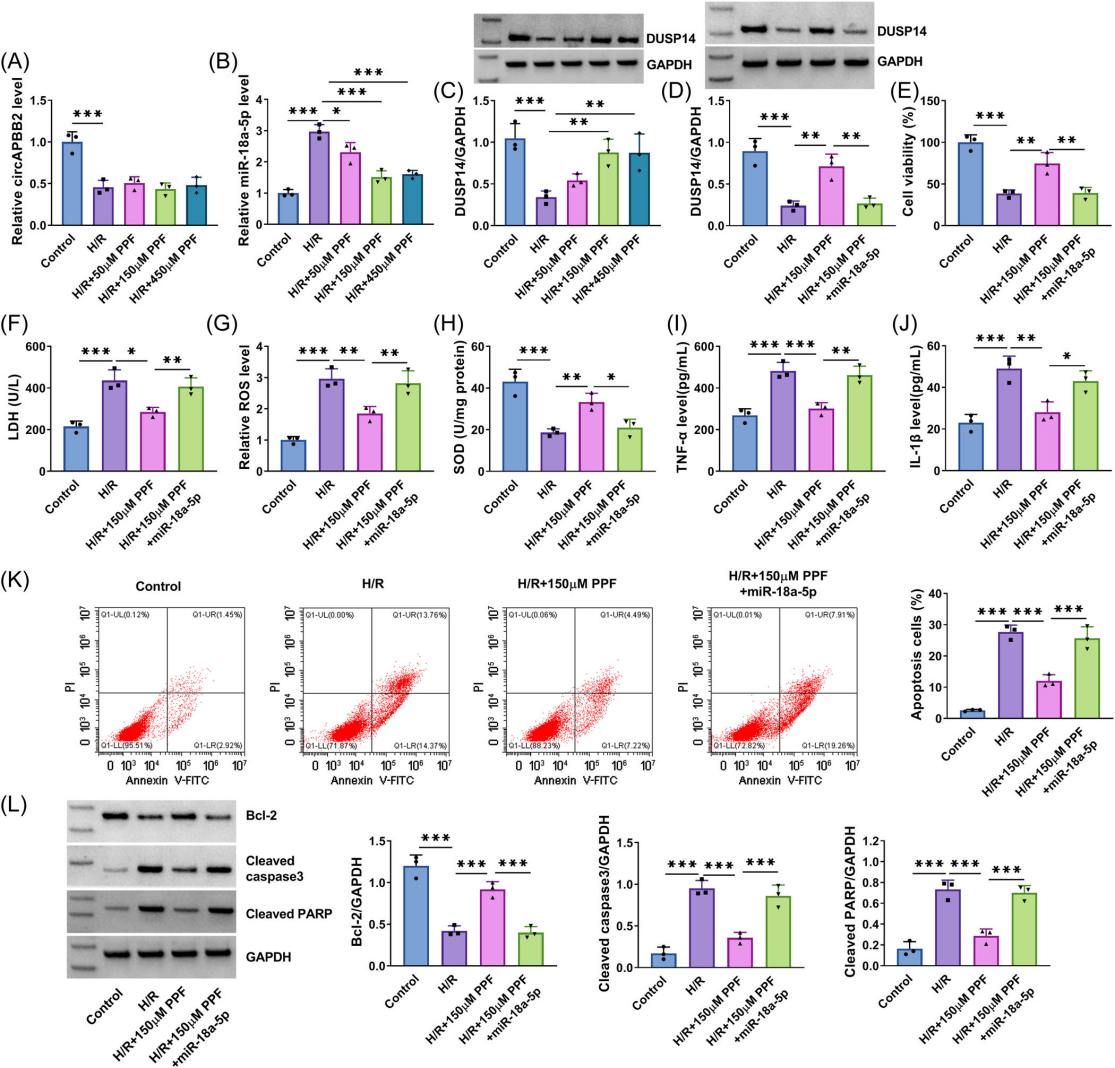
PPF treatment protected against H/R‐induced myocardial cell injury through miR‐18a‐5p. (A, B) The effects of PPF and H/R treatments on circAPBB2 and miR‐18a‐5p expression were analyzed by qRT‐PCR (*N* = 3). (C) The effects of PPF and H/R treatments on DUSP14 protein expression were analyzed by Western blot analysis assay (*N* = 3). AC16 cells were divided into H/R group, H/R + 150 μM PPF group, H/R + 150 μM PPF+miR‐18a‐5p group, with AC16 cells with normal treatment as controls. DUSP14 protein expression was analyzed by Western blot analysis assay (D) (*N* = 3). Cell viability was assessed by CCK‐8 assay (E) (*N* = 3). LDH, ROS and SOD levels were detected by commercial detection kits (F−G) (*N* = 3). TNF‐α and IL‐1β levels were quantified by ELISAs (I, J) (*N* = 3). Cell apoptosis was quantified by flow cytometry assay (K) (*N* = 3). Cleaved caspase 3, cleaved PARP and Bcl‐2 expression were detected by Western blot analysis (L) (*N* = 3). *p < .05, **p < .01 and ***p < .001. Significant differences were determined using one‐way analysis of variance with Tukey's test. Bcl‐2, B‐cell lymphoma‐2; CCK‐8, cell counting kit‐8; H/R, hypoxia/reoxygenation; IL‐1β, interleukin‐1β; LDH, Lactate dehydrogenase; ROS, reactive oxygen species; SOD, superoxide dismutase; TNF‐α, tumor necrosis factor‐α.

The data of Figure 5A revealed that H/R treatment significantly decreased circAPBB2 expression, but the effect was not affected after stimulation of various concentrations of PPF (50, 150, and 450 μM). As shown in Figures 5B,C, H/R treatment increased miR‐18a‐5p expression and decreased DUSP14 expression, whereas these effects were relieved after PPF stimulation. According to the results of Figures 5B,C, 150 μM of PPF was used in the following studies. H/R treatment‐induced inhibition in DUSP14 expression and cell viability was attenuated after PPF stimulation, but these effects were rescued after transfection with miR‐18a‐5p mimics (Figures 5D,E). The increased LDH and ROS production and decreased SOD activity by H/R treatment were attenuated after PPF stimulation; however, these effects were restored by miR‐18a‐5p mimics (Figure 5F−H). The results also revealed that H/R treatment‐induced promotion in TNF‐α and IL‐1β levels was relieved after the addition of PPF, but these effects were rescued after transfection with miR‐18a‐5p mimics (Figures 5I,J). As expected, PPF treatment relieved the promoting effects of H/R stimulation on AC16 cell apoptosis and cleaved caspase 3 and cleaved PARP expression and its inhibitory effect on Bcl‐2 expression, whereas these effects were rescued after miR‐18a‐5p expression was increased (Figures 5K,L).

### 3.6 PPF treatment enhanced circAPBB2‐mediated effects in H/R‐treated AC16 cells

**Figure 6:**
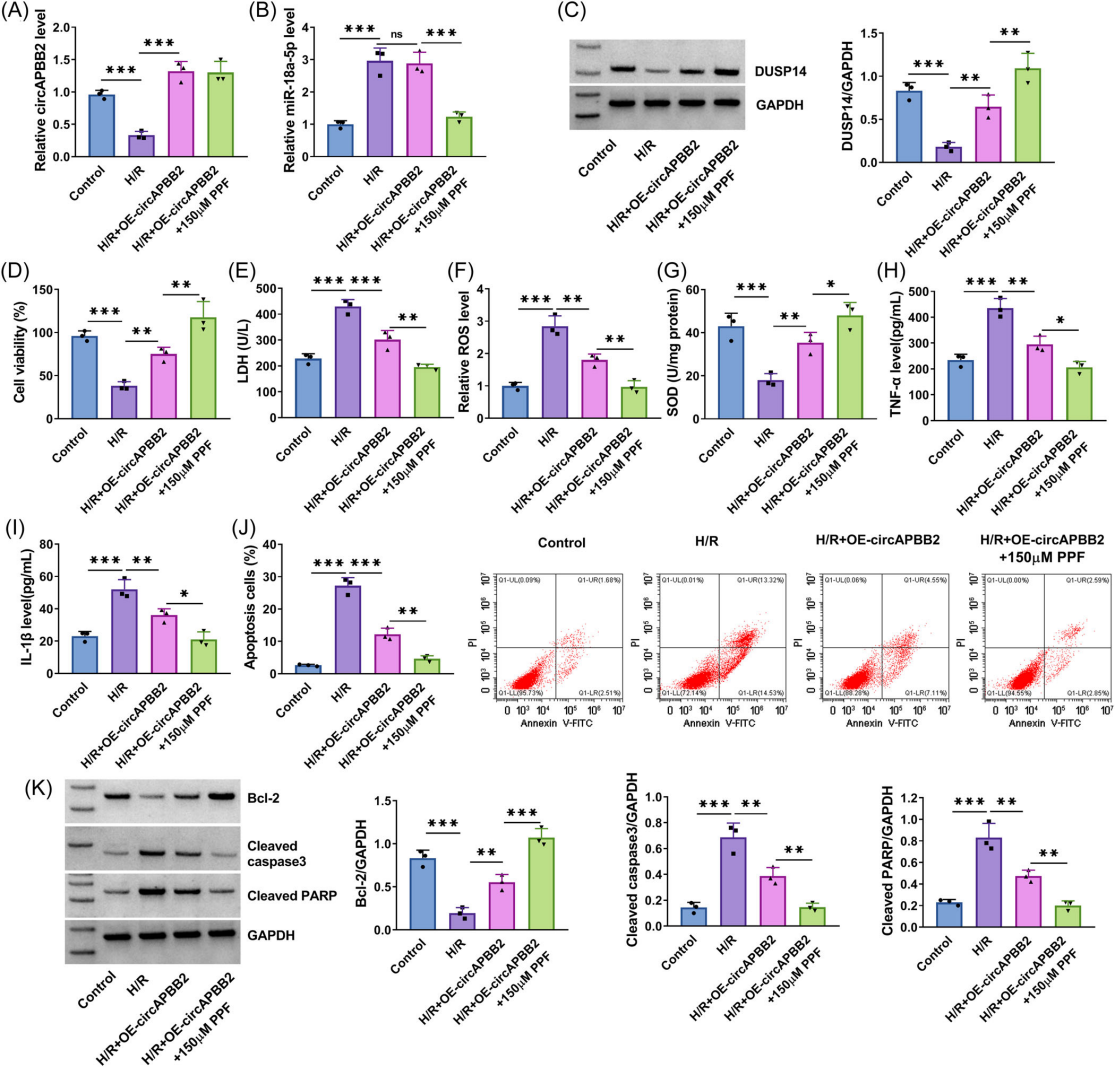
PPF treatment enhanced circAPBB2‐mediated effects in H/R‐treated AC16 cells. AC16 cells were divided into H/R group, H/R + OE‐circAPBB2 group, H/R + OE‐circAPBB2 group+150 μM of PPF group, with AC16 cells with normal treatment as controls. CircAPBB2 and miR‐18a‐5p expression were analyzed by qRT‐PCR (A, B) (*N* = 3). DUSP14 protein expression was analyzed by Western blot analysis assay (C) (*N* = 3). Cell viability was assessed by CCK‐8 assay (D) (*N* = 3). LDH, ROS and SOD levels were detected by commercial detection kits (E‐G) (*N* = 3). TNF‐α and IL‐1β levels were quantified by ELISAs (H, I) (*N* = 3). Cell apoptosis was quantified by flow cytometry assay (J) (*N* = 3). Cleaved caspase 3, cleaved PARP and Bcl‐2 expression were detected by Western blot analysis (K) (*N* = 3). *p < .05, **p < .01 and ***p < .001. Significant differences were determined using one‐way analysis of variance with Tukey's test. Bcl‐2, B‐cell lymphoma‐2; CCK‐8, cell counting kit‐8; H/R, hypoxia/reoxygenation; IL‐1β, interleukin‐1β; LDH, Lactate dehydrogenase; ROS, reactive oxygen species; SOD, superoxide dismutase; TNF‐α, tumor necrosis factor‐α.

H/R treatment‐induced inhibition of circAPBB2 expression was relieved by increasing circAPBB2 expression, but the result was not affected after PPF treatment (Figure 6A).

H/R treatment‐induced promotion of miR‐18a‐5p expression was not affected after circAPBB2 overexpression but relieved after combination treatment of circAPBB2 overexpression and PPF treatment (Figure 6B). As shown in Figure 6C, H/R treatment‐induced inhibition of DUSP14 expression was relieved by increasing circAPBB2 expression, and this effect was increased after PPF treatment. Subsequently, H/R treatment‐induced inhibition in cell viability and SOD activity and promotion in LDH and ROS production were attenuated when circAPBB2 expression was increased, and these effects were enhanced by PPF treatment (Figure 6D−G). In addition, the results showed that H/R treatment‐induced promotion in TNF‐α and IL‐1β levels was relieved by ectopic circAPBB2 expression, and the effect was promoted after PPF stimulation (Figures 6H,I). Comparatively, enforced circAPBB2 expression relieved the promoting effects of H/R stimulation on AC16 cell apoptosis and cleaved caspase 3 and cleaved PARP expression and its inhibitory effect on Bcl‐2 expression, and these effects were increased after PPF treatment (Figures 6J,K).

## 4 DISCUSSION

Myocardial infarction is defined as cardiomyocyte cell death and its progression involves the regulation of noncoding RNAs such as long noncoding RNAs, miRNAs and circRNAs.^19^, ^20^, ^21^, ^22^ Dill et al. have reported that the imprinted Dlk1‐Dio3 genomic region that contains a noncoding RNA cluster and regulates cardiac homeostasis has the potential as a therapeutic target for CVDs.^23^ Convincing evidence has indicated the contribution of circRNAs to cardiac inflammation, cardiac fibrosis, cardiac regeneration, autophagy, apoptosis, and oxidative stress signaling during myocardial infarction.^24^ The present work was the first one to report the role of circAPBB2 in human myocardial I/R injury. We found that PPF treatment synergized with circAPBB2 to protect against hypoxia‐induced cell injury through regulation of the miR‐18a‐5p/DUSP14 pathway.

Herein, we identified that circAPBB2 was formed via the head‐to‐tail splicing of the APBB2 gene, and its sequence was relatively conserved, as determined by the RNase R treatment assay. We also determined the cytoplasmic localization of circAPBB2. Subsequent results revealed that hypoxia treatment‐caused cytotoxicity, oxidative stress and inflammation were attenuated by increasing circAPBB2 expression in AC16 cells. Bcl‐2 gene has an important role in mediating disease progression via regulating proapoptotic and antiapoptotic signal pathways.^25^ The caspase family is a key driver of apoptosis, and caspase‐3 is an executioner in the apoptosis process.^26^ PARP is a preserved nuclear enzyme, and its cleavage is a marker of cell apoptosis.^27^ In this work, the three apoptosis‐related proteins were selected to verify the effects of circAPBB2 on hypoxia treatment‐induced AC16 cell apoptosis. The results showed that H/R treatment induced AC16 cell apoptosis, decreased Bcl‐2 production and increased cleaved caspase‐3 and cleaved PARP expression; however, ectopic circAPBB2 expression rescued the hypoxia treatment‐caused effects. Thus, circAPBB2 may be a novel protective gene against cardiomyocyte damage. Moreover, the results showed that PPF synergized with circAPBB2 to protect AC16 cells against H/R‐induced damage, indicating the therapeutic potential of PPF combined with circAPBB2 for myocardial I/R injury.

MiRNAs play essential roles in CVDs by regulating cell death, cell proliferation, inflammation, and angiogenesis.^28^ Zhang et al. have revealed that miR‐125b targets BCL2 antagonist/killer 1 to inhibit cardiomyocyte apoptosis after heart failure.^29^ MiR‐29a downregulation ameliorated myocardial IR injury by regulating sirtuin 1 and inhibiting oxidative stress and pyroptosis.^30^ Cardiac remodeling after myocardial infarction also involves the regulation of miRNAs.^31^ Additionally, cortical bone derived stem cells repair the heart after myocardial injury, and the underlying mechanism also involves miRNAs.^32^ Increasing studies support that circRNAs act as miRNA sponges in regulating various biological processes.^33^ Our work determined the role of circAPBB2 as a miR‐18a‐5p sponge. Previous data suggested that patients with high miR‐18a‐5p expression were associated with cardiovascular events.^34^ Moreover, miR‐18a‐5p expression was increased in H/R‐treated cardiomyocytes when compared with the control group, and transfection of miR‐18a‐5p mimics relieved isoflurane‐induced promoting effect on cell apoptosis.^35^ Consistently, our data confirmed its upregulation in hypoxic cardiomyocytes. Rescue assays demonstrated that miR‐18a‐5p inhibitors ameliorated hypoxia‐induced cytotoxicity, oxidative stress, inflammation and apoptosis of AC16 cells. In addition, the protective effect of circAPBB2 and PPF treatment against hypoxia‐induced AC16 cell damage involved miR‐18a‐5p downregulation.

MiRNA targets are the major signaling molecule core in the pathogenesis of cardiac fibrosis.^36^ The present work confirmed that DUSP14 was the miR‐18a‐5p downstream target. The DUSP family dephosphorylates DUSPs' substrates at tyrosine residues and is a key molecular regulator, serving key parts in antiobesity responses and immune responses.^37^ DUSP14, a member of the DUSP family, can protect the heart against cardiac failure.^38^ A study reported in 2018 has confirmed that DUSP14 expression is inhibited after cardiac I/R injury and that its silencing contributes to cardiac I/R damage.^38^ Consistently, we reported its downregulation in H/R‐induced cardiomyocytes. Moreover, the protective effects of miR‐18a‐5p inhibitor against cardiomyocyte damage involved DUSP14 upregulation. DUSP14 negatively modulates the TCR signaling pathway, and its silencing enhances ERK activation.^39^ The ERK pathway inhibits endoplasmic reticulum stress‐induced protective effect against cardiomyocyte injury.^40^ Thus, DUSP14 ameliorates cardiomyocyte damage through the ERK pathway. Our evidence also demonstrated that H/R‐induced DUSP14 expression inhibition could be regulated by the circAPBB2/miR‐18a‐5p axis. The above results could partially explain how circAPBB2 and PPF regulated hypoxia‐induced cardiomyocyte damage.

In conclusion, our results demonstrated that PPF synergized with circAPBB2 to protect AC16 cells against H/R‐induced damage through the circAPBB2/miR‐18a‐5p/DUSP14 pathway. The present work was the first one to report the involvement of circRNA in PPF‐induced protective effect on cardiomyocyte damage after myocardial I/R. However, this work has several limitations. Although cardiomyocyte apoptosis, inflammation and oxidative stress are essential features in cardiomyocyte damage, these cell biological behaviors cannot completely represent cardiomyocyte injury. In addition, we only conducted the study using AC16 cells, which cannot fully replicate the events that occur during myocardial I/R injury. Future preclinical studies should examine the effectiveness of the novel mechanism in animal models to confirm that these effects are maintained. Nevertheless, the present work offers a potential strategy to prevent myocardial I/R damage. PPF combined with circAPBB2 may ameliorate myocardial I/R injury.

## AUTHOR CONTRIBUTIONS

**Chenghao Jin**: Conceptualization; data curation; resources; software; validation; writing—original draft. **Shunnv Yuan**: Data curation; investigation; methodology; software. **Longyi Piao**: Formal analysis; investigation; resources; supervision; validation. **Mingcheng Ren**: Investigation; methodology; resources; software. **Qiang Liu**: Data curation; formal analysis; methodology; visualization.

## CONFLICT OF INTEREST STATEMENT

The authors declare no conflict of interest.
